# Key questions about aortic insufficiency in patients with durable left ventricular assist devices

**DOI:** 10.3389/fcvm.2022.1068707

**Published:** 2022-11-24

**Authors:** Eliza Calin, Anique Ducharme, Michel Carrier, Yoan Lamarche, Walid Ben Ali, Pierre-Emmanuel Noly

**Affiliations:** ^1^Department of Surgery, Montreal Heart Institute, Université de Montreal, Montreal, QC, Canada; ^2^Department of Medicine, Montreal Heart Institute, Université de Montreal, Montreal, QC, Canada

**Keywords:** durable left ventricular assist devices, aortic insufficiency, valvular heart disease, pathophysiology, heart failure, mechanical circulatory support (MCS)

## Abstract

The development of the latest generation of durable left ventricular assist devices (LVAD) drastically decreased adverse events such as pump thrombosis or disabling strokes. However, time-related complications such as aortic insufficiency (AI) continue to impair outcomes following durable LVAD implantation, especially in the context of long-term therapy. Up to one-quarter of patients with durable LVAD develop moderate or severe AI at 1 year and its incidence increases with the duration of support. The continuous regurgitant flow within the left ventricle can compromise left ventricular unloading, increase filling pressures, decrease forward flow and can thus lead to organ hypoperfusion and heart failure. This review aims to give an overview of the epidemiology, pathophysiology, and clinical consequences of AI in patients with durable LVAD.

## Introduction

Durable left ventricular assist device (LVAD) therapy is indicated in selected patients with advanced heart failure refractory to guideline-directed medical treatment to improve survival and quality of life. Improvement of the technology with the latest generation of pump (continuous-flow, fully magnetic) and the modifications in the cardiac allocation system in the United States contributed to significantly change the landscape of indications and outcomes in patients with durable LVAD (1). Most patients are now implanted as destination therapy or bridge to candidacy (2). Survival following LVAD implantation has reached 90% (3) and 58.4% at 1 and 5 years (4). Although a significant reduction in adverse events such as pump thrombosis or stroke is observed, time-related adverse events such as aortic insufficiency (AI) remain an area of concern in the context of increased support duration (5). The presence of significant AI in patients with durable LVAD can compromise the functional and survival benefit of the therapy. Understanding the pathophysiology and the hemodynamic consequence of AI is critical to improve patient’s management and to optimize outcomes following LVAD implantation. The purpose of this comprehensive review is to describe the pathophysiology, hemodynamic and clinical consequences of AI in patients with durable LVAD.

## Epidemiology of aortic insufficiency in patients with durable left ventricular assist devices

### What is the prevalence of aortic valve disease before durable left ventricular assist device implantation?

Approximately 5% of patients who were evaluated for a LVAD or heart transplantation suffered from moderate or severe AI (Table 1) (6–8). In a sub-analysis of the Multicenter Study of MagLev Technology in Patients Undergoing Mechanical Circulatory Support Therapy with HeartMate-3 (MOMENTUM-3) trial portfolio, 27% of the 1,790 patients who received a HeartMate-3 exhibited some degree of AI, with 2.3% having moderate or severe AI; amongst this group, 95% underwent an aortic valve procedure at the time of LVAD implantation (8).

**TABLE 1:** Summary of aortic insufficiency (AI) prevalence pre-left ventricular assist device (LVAD) implant, during left ventricular assist device (LVAD) support and clinical impact.

Study (Year of cohort)	Study type	*N*	Prevalence	AI severity	Clinical impact AI vs. no AI groups
**Prevalence before LVAD**					
Pal et al. (6) (2005–2007)	Retrospective analysis of HMII BTT trial (multicentre prospective cohort)	251	4.8%	Severe	–
Robertson et al. (7) (2006–2012)	Observational, retrospective/prospective	5,344	3.9%	Moderate or severe	–
John et al. (8) (2014–2016)	Sub-analysis of prospective, multicentre, randomized clinical trial	1,790	2.3% 26.8%	Moderate or severe Any AI	–
Tanaka et al. (9) (2006–2018)	Observational, retrospective	604	18.4%	Mild or greater	No survival difference Higher readmissions in AI group (<0.01)
**Prevalence of AI during LVAD support**					
Hiraoka et al. (11) (2005–2012)	Observational, retrospective	82	52%	More than mild	–
Truby et al. (12) (2006–2016)	Retrospective analysis of INTERMACS study	10,603	13.2%	Moderate to severe	Higher mortality (*p* < 0.005) and readmissions (*p* < 0.015) in moderate-severe AI group
Kagawa et al. (13) (2004–2018)	Observational, retrospective	316	No AI: 5.5% Trace AI: 13.9% Mild AI: 37.6%	More than mild, at 1 year	Higher mortality in significant AI group (*p* = 0.06)
Pak et al. (15) (2004–2009)	Observational, retrospective	HMI 93 HMII 73	HMI: 11.1% HMII: 24.8%	Mild to moderate or greater, at 1 year	–
Aggarwal et al. (16) (2005–2011)	Observational, retrospective	79	52%	Mild or greater, at a median follow-up of 187 days	Higher mortality in AI group (*p* = 0.03). No difference in readmissions
Jorde et al. (17) (2004–2013)	Observational, prospective, and retrospective	224	22.4%	Mild or greater, at 1 year	–
Cowger et al. (18) (2000–2011)	Observational, prospective	166	36%	Mild to moderate or greater, at 1 year	No difference in 2 year survival
Rajagopal et al. (10) (2004–2011)	Observational, retrospective	184	11.4%	Moderate or greater	No difference in survival
Deo et al. (19)	Systematic review	657	25%	- Support period 412 days	No difference in survival
Holley et al. (20) (2005–2013)	Observational, retrospective	237	15.2%	Moderate or severe	No difference in overall survival at 1 year

Progression of AI severity, in patients with pre-existing AI at the time of LVAD implant, has been reported in several publications (9–13). Patients with mild AI before LVAD implantation progress to significant AI at a higher rate than those who had trace or no AI. In a study by Kagawa et al. 94.5% of patients with no AI pre-operatively were free from significant (more than mild) AI at 1 year in comparison to only 62.4% in the group with mild AI pre-operatively (13).

### What is the prevalence of aortic insufficiency during left ventricular assist device support?

Between 11 and 52% of patients develop *de novo* AI on LVAD support (Table 1) (9, 14–19). The frequency of AI progressively increases with time. In a cohort of 78 patients implanted with a HeartMate-XVE (*n* = 25) or a HeartMate-II (*n* = 53) between 2004 and 2008, Cowger et al. found that 11% of these patients presented with moderate to severe AI at 6 months, 26% at 1 year and 51% at 18 months (14). Noteworthy, these numbers represent the data of both pulsatile (HeartMate-XVE) and continuous flow (HeartMate-II) devices. Patients receiving a HeartMate-II had more progressive AI than those receiving the HeartMate-XVE (14). Another more contemporary study of patients implanted only with non-pulsatile devices showed a lower rate of AI, where the freedom from moderate or severe AI at 1,3 and 5 years was 94%, 76%, and 65%, respectively in a cohort of 237 patients (20).

### What are the risk factors associated with *de novo* aortic insufficiency in patients with durable left ventricular assist devices?

The most important risk factors associated with development or progression of AI in LVAD patients include older age, sex (female), absence of aortic valve opening, smaller body surface area and longer LVAD support duration (9, 12, 14, 19, 21). A correlation between a smaller aortic root diameter and development of AI has also been shown and might explain the higher incidence *de novo* AI in females (22). Surgical factors, such the location and the angulation between the outflow graft and the ascending aorta also play a role (22). It has been observed that the most desirable anastomosis site should be 2 cm above the sinotubular junction at an angle ≥90° transversally and between 60° and 120° in the coronal plane (23, 24). Because AI develops with time, the destination therapy strategy is associated with a higher rate of AI compared to the bridge to transplant strategy. Finally, continuous flow pumps seem to generate more AI than pulsatile pumps (14, 19). Tanaka et al. have demonstrated that pre-implant mild or greater AI and longer LVAD support were risk factors for moderate or greater AI post-LVAD (9). Other pre-operative characteristics such as hypertension, diabetes, dyslipidemia, and the left ventricular (LV) ejection fraction have not been associated with AI (21).

## Pathophysiology of aortic insufficiency in patients with durable left ventricular assist devices

### What are the histopathological findings in the aortic valve in left ventricular assist device supported patients with aortic insufficiency?

Left ventricular assist device support can lead to AV fusion (25). The precise cause of aortic valve commissural fusion is still unknown. Some authors describe leaflet thickening on the aortic side while others have noted a thinning and shortening of leaflets (26–28). It is speculated that fusion is caused by extended time of leaflet coaptation due to the little to no antegrade flow through the valve (25, 29). Possible mechanisms include morphological changes in valvular endothelial cells under different shear strains or an environment that is completely static and encourages local fibrosis (25, 29). When the valve is closed, strong transvalvular pressures (TVP) cause the valve leaflets to stretch. As the leaflets open, they loosen up. Because higher TVP are applied to the leaflets with LVAD use, in a constant fashion as opposed to intermittently, collagen synthesis and remodeling are stimulated (25, 30).

Stasis develops on the ventricular surface of the valve when the AV remains closed and thus promotes thrombus formation and organization, which furthers leaflet fusion (14, 25, 29, 30). Wang et al. state that the leaflet fusion can be responsible for the retraction of the leaflet tips and the generation of a central orifice that becomes fixed in the absence of intermittent AV opening, causing AI (25).

### How can a durable left ventricular assist device induce aortic insufficiency?

The mechanisms of AI are multifactorial. The absence of aortic valve (AV) opening is one of the strongest factors associated with AI. Durable LVAD promote LV unloading by pumping the blood from the LV directly into the aorta, which decreases LV pressures. The transvalvular gradient is defined as the difference in pressure between the aortic root and the LV. With an LVAD, the transvalvular gradient is increased due to the unloaded LV and the elevation of the pressure in the aorta by the continuous flow from the outflow graft (30). This contributes to the closure of the AV (30). The increased load on the AV causes valve deterioration and remodeling, which results in AI (30).

### How does a left ventricular assist device change the aortic root biomechanics?

As described by John et al., normal valve biomechanics are dependent on the distensibility of the sinus tissue and the pressure cycle in the aortic root, pressure pulsatility and vortex generation (30). The retrograde flow from the LVAD prevents vortices from forming, resulting in early valve closure and a shortened systole. Thrombus formation can be found more frequently in the non-coronary sinus despite the wash out provided by the retrograde flow, due to increased blood stagnation secondary to the absence of coronary arteries draining that particular sinus (30).

Left ventricular assist device support can also contribute to the development of aortic root dilation and can thus participate to AI. The underlying mechanism used for aortic root dilation in LVAD patients seems to be the increased aortic wall sheer stress caused by the turbulence induced by the device (14, 31, 32). This leads to thinning of the aortic wall by apoptosis of smooth muscle cells and by a decrease in elastin content (14, 31, 32). In fact, aortic root diameters tend to be larger at baseline and at follow-up for patients who develop AI during LVAD support as opposed to those without AI (15). Fine et al. noted a small increase in aortic root diameter in the first 6 months post-LVAD implant which was associated with AI development, but aortic diameters remained stable thereafter (31). On the contrary, some authors have found an increase in aortic wall thickness, collagen, or smooth muscle content (33).

## How to assess aortic insufficiency severity in left ventricular assist device patients?

First, it is important to evaluate whether there is opening of the AV or not, using the M-mode in the parasternal long axis view, over 10 cardiac cycles (34). Then, Color Flow Doppler is added to semi-quantify the severity of the AI and its timing during the cardiac cycle. Of note, the echocardiographic evaluation and quantification with conventional methods (i.e., vena contracta, jet width/left ventricular outflow tract diameter, pressure half-time, and proximal iso-velocity surface area) is more difficult in LVAD patients with AI due to the presence of multiple eccentric jets and acoustic shadow caused by the device (35). The volumetric assumptions used to derive those formulas are incorrect in this clinical setting, as AI on LVAD occurs throughout the cardiac cycle, both in systole and diastole (5).

Therefore, new methods have been described for the evaluation of AI in LVAD patients: diastolic flow acceleration and the systolic-to-diastolic (S/D) velocity ratio of the outflow cannula (35).

A detailed description of all the available methods is beyond the scope of this review and can be found elsewhere (5). Briefly, from a modified right parasternal view, a Pulse Wave Doppler is placed in the outflow cannula, <1 cm proximal to its anastomosis to the ascending aorta. Diastolic acceleration is calculated by measuring the diastolic slope, from the beginning to the end of diastole; the S/D ratio is obtained by dividing the peak systolic velocity by the peak end-diastolic velocity (35). This S/D velocity ratio is inversely proportional to the severity of AI, and the diastolic acceleration of the outflow cannula is directly proportional with the severity of AI (34, 35). Moderate or greater severity AI, defined as a regurgitant fraction >30%, will exhibit a S/D ratio of <5.0 or a diastolic acceleration of >49.0 cm/s^2^ (5, 35).

By using these methods, Grinstein et al. reclassified approximately 30% of patients with trace/mild AI as evaluated by conventional methods to at least moderate AI (34). Patients who were diagnosed with more than moderate AI using these new TTE parameters had a higher PCWP than patients who had less severe AI. Additionally, there was a non-significant trend toward declining right ventricular (RV) function in patients with moderate or higher levels of AI as determined by these updated TTE criterias (34). However, there was no such difference when AI was evaluated using conventional TTE parameters (34).

## Hemodynamic consequences of aortic insufficiency in patients with durable left ventricular assist devices

### What are the hemodynamic changes in patients with durable left ventricular assist devices and aortic insufficiency?

**FIGURE 1:**
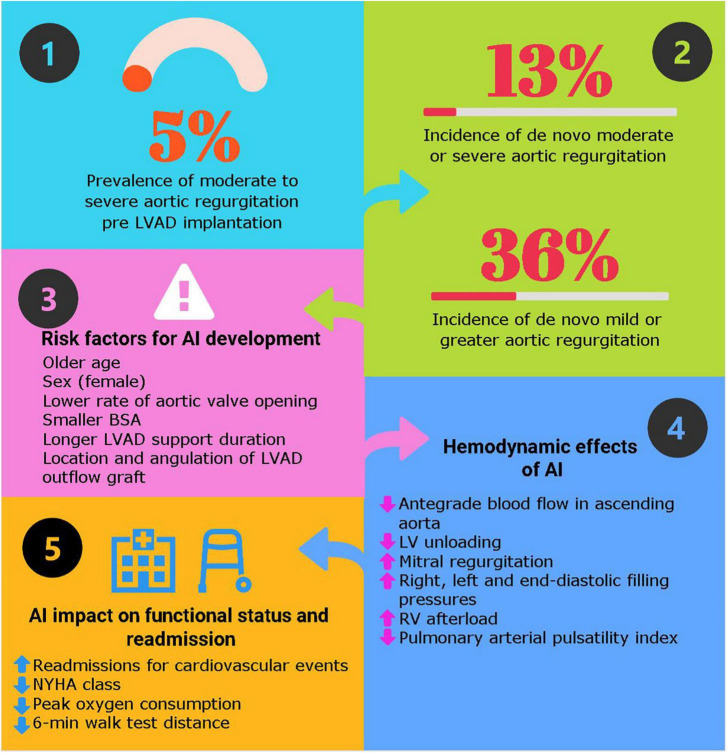
Key answers about aortic insufficiency in patients with durable left ventricular assist device. Hemodynamic effects of left ventricular assist devices (LVAD) with aortic insufficiency. Adapted with permission from Noly et al. (53).

When AI is hemodynamically significant, the blood circulates in a “closed loop” between the pump, the aortic root, and the LV (Figure 1). As the proportion of retrograde flow increases, sub-optimal LV unloading occurs, resulting in increased left-sided filling pressures and volume overload to the LV. These hemodynamic changes associated with AI result in an increase of the left ventricular end-diastolic diameter, reduced systolic blood pressures, cardiac output and elevations in brain natriuretic peptide levels, when compared with patients with no/mild AI (12, 36).

This has been nicely demonstrated by Sayer et al., where AI initially causes increased biventricular filling pressures [central venous pressure (CVP) and pulmonary capillary wedge pressure (PCWP)] while maintaining the same cardiac index (CI) (36). With time, if the PCWP remains elevated, pulmonary hypertension develops causing additional strain on the RV (36).

### What are the consequences of aortic insufficiency on right ventricular function?

The right ventricle (RV) remains the Achilles heel of this technology. Right-sided failure can occur after LVAD implantation, when a vulnerable RV faces a sudden rise in cardiac output provided by the LVAD, and is unable to accommodate to this increased preload. In addition, altered RV contractility secondary to the withdrawal of inotropes or to the loss of septal contraction may contribute (37). Aggressive unloading of the LV by the pump may cause an interventricular septal shift toward the LV, altering the RV geometry and its contractility (38).

The presence of AI can further compromise RV function, indirectly through its impact on increased LV filling pressures and reduced effective pre-load. First, higher pulmonary wedge pressures lead to a passive rise in pulmonary artery pressures and consequently higher RV afterload (39). In addition, the closed-loop circuit described above creates a reduction in the effective cardiac output, thus reducing RV pre-load and potentially contributing to RV failure.

In patients with significant pulmonary hypertension before LVAD implantation, it may not totally resolve post-operatively despite LV unloading by the device, thereby leaving some residual and variable degree of increased RV afterload (40). These patients may be more susceptible to suffer from RV failure in the presence of AI; indeed, Sayer et al. demonstrated the impact of AI on a decreasing pulmonary artery pulsatility index (PAPI) (6).

## Clinical implications of aortic insufficiency in patients with durable left ventricular assist devices

### Impact of aortic insufficiency on mortality?

The impact of AI on mortality remains controversial. Some authors reported a higher mortality rate in patients with AI (12, 13) while others do not (9, 16, 18–20, 41). Kagawa et al. and Truby et al., reported higher mortality rates amongst patients with significant (≥moderate) AI, 59.5% vs. 37.2% (*p* = 0.006) and 28.6% vs. 22.8% (*p* = 0.05), respectively (12, 13). This discrepancy might be explained by the presence of more severe AI in the papers having found a mortality difference as compared to the ones who have not. Another possible explanation is that some studies might be underpowered to detect such a difference due to their small cohorts. In contrast, the study by Truby et al., is one of the largest studies published on the subject, with over 10,000 patients and thus plays a very important role (12).

### Functional status, hospital readmission, adverse events?

Aortic insufficiency in LVAD patients leads to worsening functional status and higher readmission rates as opposed to patients with a competent valve (9, 12, 13, 41). When comparing patients without AI and those with mild AI at the time of LVAD implantation, patients with mild AI had a worse NYHA class and more readmissions caused by heart failure (HR 2.62, *p* < 0.01) (9). The survival was similar between groups, over a short follow-up of 3 years (9). Similarly, Imamura et al. found that at 6 months following LVAD implantation, patients with mild AI showed reduced peak oxygen consumption during cardiopulmonary exercise tests compared to those without AI (11.0 ± 3.3 vs. 14.4 ± 3.5 ml/min/kg^–1^, *p* = 0.004) and a shorter 6-min walk distance (328 ± 84 vs. 407 ± 66 m, *P* = 0.001) (41). During the 2-year LVAD support period, patients with mild or greater AI had a greater readmission rate for cardiovascular events than patients without AI (55% vs. 8%, *p* = 0.001) (41).

The impact of AI on post-transplant outcomes in patients supported with LVAD is not known. Although the duration of LVAD support is not associated with post-transplantation outcomes, it is reasonable to postulate that increased pulmonary pressures might lead to higher rates of primary graft failure secondary to pulmonary hypertension, (42). RVAD or various post-operative complications and end-organ damage (acute kidney injury, hepatic congestion resulting in bleeding, and inflammatory syndrome). This hypothesis remains to be tested.

## How could we prevent *de novo* or worsening aortic insufficiency in patients supported with left ventricular assist devices?

### Medication optimization

One of the aims of medical management is to relieve congestive symptoms with diuretics and improve filling pressures with vasodilators (18). In addition to blood pressure control, vasodilators decrease aortic wall stress and thus may limit progressive aortic dilation (18). The International Society of Heart and Lung Transplantation guidelines recommend a mean arterial pressure goal <80 mmHg (43). While a combination of many classes of agents may be necessary to achieve adequate blood pressure control, including beta-blockers, angiotensin-converting enzyme inhibitors, angiotensin receptor blockers, and diuretics, there are evidences that the Guideline-directed medical therapy should be pursued in LVAD-patients. In cases of refractory heart failure, inotropes may be necessary.

### Pump parameters optimization

Targeting pump speeds in the lower range may be helpful to promote AV opening and ultimately reduce the risk of developing AI. This strategy could facilitate intermittent aortic valve opening, reduce AV malcoaptation and fusion and thus prevent AI development (5, 18). This has also been suggested in cases of asymptomatic AI. The benefits of aortic valve opening must be weighed against the risk of organ hypoperfusion, as well as pump thrombosis due to low flows.

On the other hand, when congestive symptoms are present and refractory to medical therapy, patients should undergo an echocardiography guided ramp study, as well as right heart catheterization. Increasing the pump speed will initially promote LV unloading and a decrease in LV end-diastolic pressure (LVEDP). However, this will then start a vicious cycle of complete aortic valve closure leading to increased AI due to a rise in the TVP, ultimately raising the LVEDP (5, 17, 36, 44). An increase in pump speed may acutely improve CI and PCWP (36). For LVAD patients without AI, the increase in pump speed also increases the PAPI as opposed to patients with AI, where no improvement is observed (36). This may be due to the inability of the RV to increase contractility despite improved overall hemodynamics (36).

The ideal rotations per minute (RPM) are the RPM that best achieve hemodynamic optimization, defined as a PCWP <18 mm Hg, CVP <12 mm Hg and a CI >2.2 L/min/m^2^, with, ideally, intermittent AV opening and minimal mitral insufficiency (45).

When aggressive medical and pump parameters optimization fails to improve symptoms, surgical and percutaneous aortic valve interventions might be considered. The detailed description of those techniques is addressed in another article of this collection. Improvement in functional status has been observed (46, 47). Survival rates range from 55–89% at 1 year, with higher in-hospital mortality rates in the surgical group in comparison to the percutaneous group (48–52). The outcomes of these patients are based on small series; prospective validation on bigger cohorts is thus necessary. A waiting list status upgrade, for patients who are candidates for heart transplantation, may also be explored.

## Conclusion and perspective

In conclusion, the incidence of AI increases with longer support durations. Development of AI in patients supported with a durable LVAD compromises the benefit of the therapy. There is still a lack of consensus on the effect that AI has on mortality, but several studies report that AI increases heart failure related hospitalizations and contributes to the deterioration in functional status. Multiple strategies exist to minimize *de novo* AI development and its hemodynamic impact on the LV and RV during LVAD support. Further research studies are needed to better characterize the severity of AI, to better understand its impact on patients transplanted, and to prevent its development.

## Author contributions

EC was responsible for the literature review, drafting the manuscript, and the creation of the figure and table. P-EN supervised the work and provided direction. P-EN, AD, MC, YL, and WB reviewed the manuscript and provided feedback. All authors contributed to the article and approved the submitted version.
